# Temperature-Dependent Fungal Diversity, Storage Quality, and Processing Quality of High-Moisture Wheat During Post-Harvest Storage

**DOI:** 10.3390/foods15020361

**Published:** 2026-01-19

**Authors:** Yanfei Li, Zihang He, Yan Zhao, Haoxin Lv, Ge Han

**Affiliations:** School of Food and Strategic Reserves, Henan University of Technology, Zhengzhou 450001, China; liyanfei@haut.edu.cn (Y.L.); 2024930565@stu.haut.edu.cn (Z.H.); lvhaoxin0129@126.com (H.L.); hangege0921@126.com (G.H.)

**Keywords:** wheat, high-moisture, different storage temperatures, fungal, quality

## Abstract

Higher moisture content in wheat benefits processing but impairs storage stability. Current research on quality changes in high-moisture wheat under varying storage temperatures remains limited. This study systematically evaluated wheat with 14% moisture content stored at 15 °C, 20 °C, 25 °C, and 30 °C over 180 d, assessing quality parameters, mycotoxin levels, and fungal community composition. Results indicated that wheat stored at 15 °C and 20 °C maintained stable storage and processing quality. Meanwhile, the concentrations of aflatoxin B_1_, deoxynivalenol, and zearalenone in wheat across all storage temperatures remained below their respective regulatory limits of 5.00 μg/kg, 1.00 mg/kg, and 60.00 μg/kg. No visible mold appeared in wheat stored at 15 °C, 20 °C, or 25 °C for 180 d, whereas initial mold characteristics emerged at 30 °C. Fungal community analysis revealed that at 15 °C and 20 °C, the dominant genus shifted from *Bipolaris* to *Cladosporium*, while at 25 °C and 30 °C, it rapidly transitioned to *Aspergillus*. Although fungal richness showed no significant differences, diversity indices varied notably across storage temperatures. These findings provide a theoretical basis for the safe storage of high-moisture wheat.

## 1. Introduction

Wheat (*Triticum aestivum* L.) is one of the major cereal crops worldwide, with an annual global production reaching approximately 761 million tons and a cultivation area of about 217 million hectares in 2020, reflecting its wide cultivation range [1]. It provides various nutrients for humans, including carbohydrates and proteins [2]. In recent years, with the improvement of people’s health awareness, the consumption of wheat as a whole grain has increased significantly [3].

Currently, diverse methods are employed for wheat storage, including traditional storage [4], low-temperature grain storage [5], and nitrogen temperature regulation [6]. However, due to limitations in the airtightness of warehouses and related costs, low-temperature grain storage remains the predominant approach in most buildings [7]. The temperature within the grain pile ecosystem exhibits fluctuations driven by both seasonal ambient temperature variations and internal warehouse thermal conditions [8]. Failure to implement timely temperature regulation may result in localized heat accumulation, potentially causing irreversible quality deterioration and economic losses [9]. Therefore, low-temperature grain storage is of paramount importance.

Temperature is one of the most critical factors influencing quality changes in wheat during storage. High temperatures (30–60 °C) accelerate wheat respiration, leading to nutrient loss and promoting the growth of pests and molds, thereby causing quality degradation [10]. For instance, a temperature of 45 °C led to an 18.5% reduction in total available lysine and a 4% decrease in total soluble sugar [11]. Low-temperature storage (15 °C and 20 °C) effectively preserves wheat processing quality and biochemical stability [12]. Sharma et al. demonstrated that as storage temperature rises, heat stress leads to an increase in the activity levels of antioxidant enzymes in wheat [13]. On the other hand, the α-amylase in wheat is thermally inactivated at 30 °C, reducing the quality of wheat [14]. Protein component analysis showed that low-temperature storage maintained glutenin macropolymer (GMP) content at 89.4 ± 3.2% of the initial value, whereas only 72.1 ± 5.7% remained in the ambient temperature group, which was positively correlated with the disulfide bond cross-linking retention rate (r = 0.83, *p* < 0.01) [15]. Consequently, temperature variations significantly affect wheat quality indicators, including mold growth, fatty acid values, enzymatic activities, and protein composition. Low-temperature (15 °C and 20 °C) storage effectively preserves the initial quality of wheat flour.

Moisture content critically influences grain quality preservation during storage [16]. Grains with elevated moisture exhibit significantly higher susceptibility to fungal contamination compared to those below 12% moisture content [17,18]. Elevated moisture content in wheat accelerates respiratory activity and fungal proliferation, resulting in dry matter loss and quality deterioration [19]. Kibar et al. demonstrated that increasing wheat moisture content from 13% to 19% during 3-month storage at 30 °C elevated free fatty acid (FFA) content by 80%, with lipoxygenase activity accelerating rancidity [20]. Concurrently, wheat seed germination rate declined with prolonged storage and higher moisture content [21]. Cetiner et al. reported that elevated moisture levels in wheat contributed to the formation of a more stable gluten network in flour, thereby enhancing the volume and texture of baked products [22]. It has been reported that maintaining wheat at a suitably higher moisture content improved its overall quality and enhanced the viscoelasticity of the resultant flour [23]. Concurrently, flour ash content decreased marginally with moisture elevation, with hard wheat exhibiting more pronounced advantages under high-moisture conditions. In addition, low-moisture wheat products, such as dry noodles and fried instant noodles, exhibited considerable storage stability exceeding six months, while lipid oxidation emerged as their primary limitation during long-term storage [24,25]. Consequently, achieving optimal balance between wheat storage stability and processing quality remains a critical challenge.

Although some studies had elucidated metabolic profile alterations of metabolites during low-moisture wheat storage, there were few studies on systematic investigations including storage quality, processing quality, and storage microorganisms of wheat grains with 14% moisture content at different temperatures. This study systematically examines quality changes in high-moisture wheat (14% water content) during storage under different temperature regimes. The results provide a foundation to maintain high-moisture wheat quality at different temperatures.

## 2. Materials and Methods

### 2.1. Material and Treatment

Wheat (Bainong 307) was harvested from Huixian County, Xinxiang City, Henan Province, in June 2022. According to the method proposed by Lutz and Coradi [26], the moisture content of wheat was calibrated to the level of 14 ± 0.1%. The wheat samples were portioned into bags and stored in climate-controlled incubators for 180 d under the following conditions: temperatures of 15 °C, 20 °C, 25 °C, and 30 °C, each with a relative humidity maintained at 65–70%. During the whole storage period, wheat samples were collected regularly every 30 d to analyze relevant indicators.

### 2.2. Fatty Acid Value (FAV)

The FAV was determined by the method described by Zhang and Liu [27], with minor adjustments. An amount of 10 g of wheat flour sample was extracted with 50 mL of benzene for 30 min. Subsequently, 25 mL of the filtrate was mixed with a 0.04% phenolphthalein ethanol indicator and titrated with a 0.01 mol/L potassium hydroxide solution. The result was expressed as mg/100 g.

### 2.3. Unit Weight

The unit weight of wheat was conducted in accordance with GB/T 5498-2013 [28]. Prior to analysis, the instrument was calibrated. Samples were poured into the grain cylinder and leveled using a straight edge. After transferring to the unit weight cylinder, mass measurements were taken. Results from duplicate determinations with ≤3 g/L variation were averaged and reported as whole numbers. The values of unit weight were expressed in g/L.

### 2.4. Germination Rate

The germination rate of wheat was conducted in accordance with GB/T 5520-2011 [29]. Germination tests were performed on 100 intact wheat kernels at 20 ± 1 °C using a two-layer saturated filter paper system. After 7-day incubation, germination was assessed by radicle emergence ≥ 2 mm. The germination rate (%) was calculated as the proportion of successfully germinated seeds relative to the total tested.

### 2.5. Catalase (CAT) Activity

The determination of CAT activity was performed using a CAT kit (Catalog No. BC0200, Beijing Solarbio Science & Technology, Beijing, China). The absorbance was recorded at a wavelength of 240 nm; one unit of CAT activity was determined as the degradation of 1 μmol of H_2_O_2_ per gram of tissue per minute, and the results were expressed as U/g.

### 2.6. Color Measurement

The color difference meter (CR-410, Metal One Co., Ltd., Tokyo, Japan) was first subjected to blank calibration following the method of Gang Qiang. The color difference meter was allowed to warm up for 10 min to stabilize the light source. Calibration was performed sequentially, starting with zero calibration followed by white calibration using a standard white calibration tile (CR-A43). The sample was then spread to completely cover the groove, and the color parameters of L*, a*, and b* values were recorded by colorimeter.

### 2.7. Determination of Pasting Properties

The determination of pasting properties of wheat was carried out in adherence to AACC method 61-02.01 using a Rapid Visco Analyzer (RVA-TM, Perten, Wuxi, China). Wheat flour was loaded into an aluminum sample cylinder containing 25 mL of distilled water for the preparation of the suspension. A viscosity change curve was constructed by executing pre-set stirring, heating, and cooling programs.

### 2.8. Water Absorption of Gluten

The determination of gluten water absorption was conducted following GB/T 5506.4-2008 [30]. An amount of 4.8 mL of 20 g/L sodium chloride solution was added to the washing chamber of the gluten meter containing 10 g of sample at a temperature of 22 ± 2 °C. After thorough washing, the wet gluten was centrifuged for 60 s and weighed as (m1). Then, the wet gluten was dried in an oven at 105 °C until a constant weight was achieved, and the mass of the dry gluten (m2) was determined. The water absorption rate of gluten was calculated by the formula; it is reported in %.

(1) Gluten water absorption (%)=[(m1−m2)/m2]×100%

### 2.9. Alpha-Amylase (α-Amylase) Activity

The α-amylase activity was determined according to the method of Etsassala et al. [31] with minor optimizations. A standard curve was constructed using maltose mass as the abscissa and absorbance as the ordinate. Wheat flour (1.0 g) was weighed to extract the crude amylase solution. Specifically, 1 mL of the crude solution was subjected to water bath treatment at 70 °C for 15 min, cooled to room temperature, and then mixed with 1 mL of 1% starch solution. The mixture was incubated in a 40 °C constant-temperature water bath for 5 min, followed by the addition of 2 mL of 1% DNS solution. After boiling in a water bath for 5 min, the solution was diluted to 20 mL, and the absorbance was measured at 540 nm. α-Amylase activity was defined as the mass of maltose released from starch per gram of tissue per minute, and the results are expressed as mg/(g·min).

### 2.10. Total Fungi Content

Total fungi content was quantified in accordance with GB 4789.15-2016 [32]. An amount of 25 g of sample aliquot was homogenized with 225 mL of sterile diluent (0.1% peptone water) to yield a 1:10 (*w*/*v*) suspension. Serial dilutions (10:1 and 10:2) of the fungal suspension were prepared, and the corresponding dilutions were pour-plated onto different plates to determine microbial load. Plates were inverted and incubated at 28 ± 1 °C for 180 d, with colony counts recorded every 30 d. The results are expressed as log CFU/g.

### 2.11. Aflatoxin B1 (AFB1) Content

The determination of AFB_1_ in wheat was performed using an ELISA kit (Cat. No. Ml036115, Shanghai Enzyme-linked Biotechnology Co., Ltd. Shanghai, China). Based on the principle of enzyme-linked immunosorbent assay (ELISA), this method involves measuring the absorbance of standards (concentration gradient: 0 ppb, 0.03 ppb, 0.06 ppb, 0.12 ppb, 0.24 ppb, 0.48 ppb) and samples at 450 nm to construct a standard curve for calculating the AFB_1_ content. The results are expressed as μg/kg.

### 2.12. Deoxynivalenol (DON) Content

The DON content in wheat was determined using an ELISA kit (Cat. No. Ml036117, Shanghai Enzyme-linked Biotechnology Co., Ltd. Shanghai, China). Based on the ELISA principle, absorbance values of standards (0, 10, 30, 90, 270, 810 ppb) were measured at 450 nm and the content exhibited as μg/kg.

### 2.13. Zearalenone (ZEN) Content

The ZEN content in wheat was determined using an ELISA kit (Cat. No. ml036116, Shanghai Enzyme-linked Biotechnology Co., Ltd. Shanghai, China). Based on the ELISA principle, absorbance values of standards (0, 0.3, 0.9, 2.7, 8.1, 24.3 ppb) and samples at 450 nm were used to construct a standard curve for calculating ZEN levels. ZEN content is expressed as μg/kg.

### 2.14. Statistical Analysis

Statistical analysis was performed using SPSS 26.0, including one-way analysis of variance. Post hoc comparison was conducted using Duncan’s multipolar test (*p* < 0.05). Data visualization was performed using Origin 2018, and the results are expressed as mean ± standard error (*n* = 3). Principal Component Analysis (PCA) and orthogonal partial least squares discriminant analysis (OPLS-DA) were performed using the r. ‘ropls’ software package (v1.6.2). Hierarchical cluster analysis (HCA) was conducted using the Python ‘Scipy’ library (v1.0.0).

## 3. Results

### 3.1. Wheat Quality Changes

**Figure 1:**
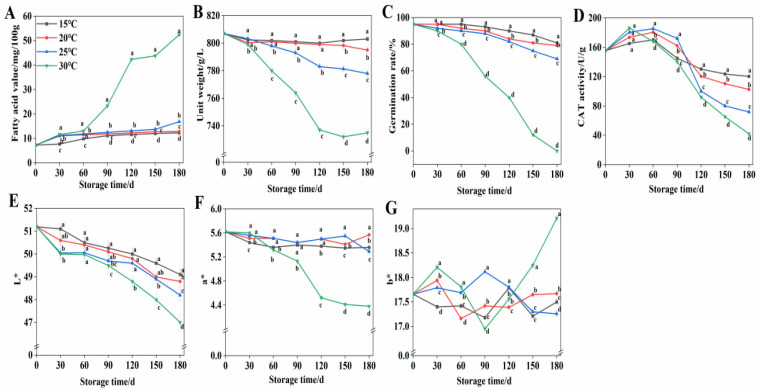
Quality changes in wheat during storage at 15 °C, 20 °C, 25 °C, and 30 °C. (**A**) Change in fatty acid value at different storage temperatures. (**B**) Changes in unit weight at different storage temperatures. (**C**) Changes in germination rate at different storage temperatures. (**D**) Changes in CAT activity at different storage temperatures. (**E**) Changes in L* values at different storage temperatures. (**F**) Changes in a* values at different storage temperatures. (**G**) Changes in b* values at different storage temperatures. Distinct letters denote statistically significant differences (*p* < 0.05).

During storage, lipid hydrolysis significantly affects the taste and quality of wheat. As shown in Figure 1A, at 15 °C, 20 °C, and 25 °C, the FAV of wheat remained relatively stable, possibly due to the inhibition of lipolytic enzyme activity as wheat adapted to the storage environment in the early stages. At 30 °C, the fatty acid values increased significantly (*p* < 0.05) during 60–120 d of storage and continued to rise at 150–180 d. Compared with 30 °C, storage at 15 °C, 20 °C, and 25 °C significantly delayed the lipid oxidation deterioration of wheat during 60–180 d. At the end of storage, the fatty acid values in wheat stored at 15 °C, 20 °C, and 25 °C decreased by 76.57%, 75.50%, and 67.89%, respectively, compared with the values of wheat stored at 30 °C.

A significant positive correlation was found between wheat unit weight and flour yield. Throughout the 180-day storage period, unit weight exhibited a consistent decline (Figure 1B). Wheat stored at 25 °C and 30 °C exhibited significantly lower unit weight compared to 15 °C and 20 °C storage conditions, reaching a minimum value of 735 g/L at 30 °C. In contrast, wheat maintained unit weight exceeding 790 g/L following 180-day storage at both 15 °C and 20 °C.

Germination rate serves as a critical quality parameter for wheat grains. Figure 1C demonstrates a progressive decline in wheat germination rates throughout the storage period. Temperatures of 15 °C, 20 °C, and 25 °C delayed the increase in germination rate, reaching 81%, 79%, and 69% higher compared with 30 °C at 180 d. Notably, at 30 °C, the germination rate remained at 90% during the initial 30 d before a significant decline ensued.

CAT plays a crucial enzymatic role in wheat storage processes. As shown in Figure 1D, after 180 d of storage, CAT activity in wheat stored at 15 °C, 20 °C, and 25 °C reached 2.86-, 2.44-, and 1.71-fold higher compared with 30 °C, respectively. Notably, at 30 °C, CAT activity peaked at 186.2 U/g on day 30, whereas at 15 °C, 20 °C, and 25 °C, the maximum activities of 170.2 U/g, 180.6 U/g, and 185.6 U/g, respectively, were observed on day 60.

Wheat color serves as a critical quality indicator during storage evaluation. As shown in Figure 1E, wheat stored at 15 °C, 20 °C, and 25 °C for 180 d exhibited L* value that were 2.1, 1.8, and 1.2 higher, respectively, compared to those stored at 30 °C. The decrease in a* value was delayed under storage at 15 °C, 20 °C, and 25 °C. After 180 d of storage, the a* value decreased by 0.26, 0.05, and 0.33, respectively, relative to the values at 0 d. At 30 °C, the a* value began to decrease markedly from day 60. After 180 d of storage, it was 0.98, 1.19, and 0.91 lower than the values recorded at 15 °C, 20 °C, and 25 °C, respectively (Figure 1F). A sharp increase in the b* value was observed in wheat stored at 30 °C after 150 d, reaching levels that were 1.71, 1.54, and 1.95 higher than those at 15 °C, 20 °C, and 25 °C by 180 d (Figure 1G).

**Figure 2:**
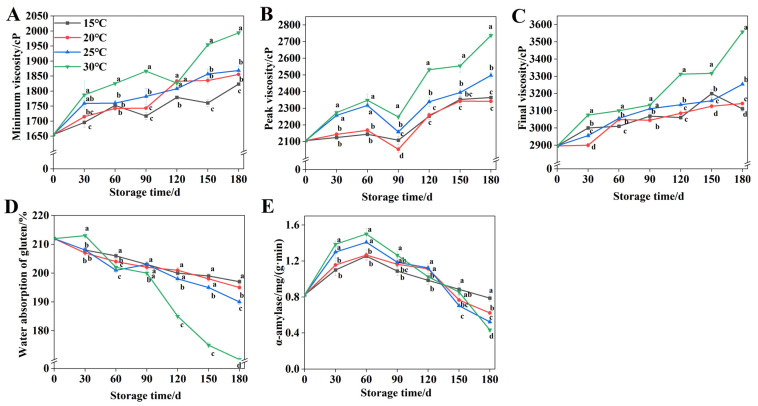
Quality changes of wheat during storage at 15 °C, 20 °C, 25 °C, and 30 °C. (**A**) Change in minimum viscosity at different storage temperatures. (**B**) Changes in peak viscosity at different storage temperatures. (**C**) Changes in final viscosity at different storage temperatures. (**D**) Changes in water absorption of gluten at different storage temperatures. (**E**) Changes in α-amylase activity at different storage temperatures. Distinct letters denote statistically significant differences (*p* < 0.05).

The gelatinization properties of wheat starch represent a critical parameter for assessing its edible and processing quality. The minimum viscosity reflects the shear resistance of wheat starch. Figure 2A demonstrates that wheat’s minimum viscosity exhibited an overall increasing trend during the 180-day storage period. At the end of storage, the minimum viscosity of wheat at 30 °C was 170.5 cP, 138.5 cP, and 125.5 cP higher than that at 15 °C, 20 °C, and 25 °C, respectively. As shown in Figure 2B, wheat peak viscosity displayed an overall increasing trend during the 180-day storage period. However, a transient decline occurred at 60 d, reaching minimal values by 90 d. Peak viscosity stabilized after 150 d of storage at 15 °C and 20 °C. At 25 °C and 30 °C, peak viscosity exhibited sustained increases beyond 90 d, attaining values of 2497.5 cP and 2737 cP, respectively, by 180 d. After 90 d of storage, the final viscosity of wheat stored at 15 °C, 20 °C, and 25 °C was lower than that of the 30 °C group. By day 180, the final viscosity at these temperatures measured 446 cP, 415.5 cP, and 303.5 cP lower, respectively, compared to the 30 °C condition (Figure 2C).

The water absorption of gluten serves as both a critical parameter for evaluating wheat storage suitability and a key indicator of wheat protein hydration properties. Figure 2D reveals an inverse relationship between storage time and water absorption of gluten in wheat, with the decline becoming more pronounced at elevated temperatures. After 120 d of storage, water absorption of gluten differed significantly (*p* < 0.05) across the four temperature conditions. After 180 d of storage, water absorption of gluten remained at 197%, 195%, and 190%, respectively., but decreased to 170% at 30 °C.

As demonstrated in Figure 2E, wheat α-amylase activity exhibited an initial increase followed by a gradual decrease throughout the 180-day storage period. The α-amylase activity of wheat peaked at 60 d of storage, reaching a maximum of 1.5 mg/(g·min) at 30 °C. From 60 to 180 d, temperature exhibited a negative correlation with enzyme activity. By the end of the storage period, the α-amylase activity in wheat stored at 15 °C, 20 °C, and 25 °C. was 1.82-, 1.44-, and 1.21-fold higher, respectively, compared with 30 °C.

### 3.2. The Pattern of Fungal Spoilage in Wheat During Storage

**Figure 3:**
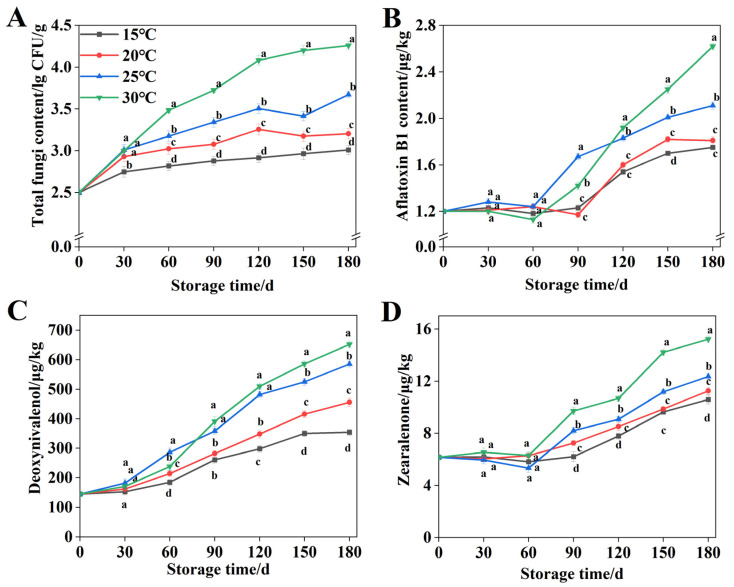
(**A**) Change in total fungi content at different storage temperatures. (**B**) Change in AFB_1_ content at different storage temperatures. (**C**) Change in DON content at different storage temperatures. (**D**) Change in ZEN content at different storage temperatures. Distinct letters denote statistically differences (*p* < 0.05).

As presented in Figure 3A, wheat exhibited a progressive increase in total fungi content across all four storage temperatures during the experimental period. The initial total fungi content in wheat grains was 2.5 log CFU/g. After 180 d of storage, the total fungal count at 15 °C and 20 °C increased by 20.4% and 28.0%, respectively, compared with 0 d, reaching 3.01 log CFU/g and 3.20 log CFU/g. In contrast, storage at 25 °C and 30 °C resulted in increases of 46.8% and 70.4% over the same period, with final counts of 3.67 log CFU/g and 4.26 log CFU/g, respectively. Furthermore, compared to storage at 20 °C, 25 °C, and 30 °C, the total fungal content at 15 °C after 180 d was reduced by 5.94%, 17.98%, and 29.51%, respectively.

AFB_1_ is a fungal secondary metabolite produced by *Aspergillus flavus* and related species [33]. As shown in Figure 2B, the initial AFB_1_ content in wheat grains was 0.025 μg/kg. At 15 °C and 20 °C, AFB_1_ content showed initial increase after 90 d of storage. At 25 °C and 30 °C, wheat exhibited earlier AFB_1_ accumulation starting from 60 d, with the most rapid increase occurring at 30 °C. The AFB_1_ content in wheat stored at 15 °C for 180 d was 3.31%, 17.06%, and 33.21% lower than compared with wheat stored at 20 °C, 25 °C, and 30 °C, respectively, with a measured value of 1.75 μg/kg.

DON is alternatively designated as vomitoxin [34]. As shown in Figure 3C, the DON content in wheat began to show significant differences among the four temperatures when stored for 60 d (*p* < 0.05). After being stored for 90 d, the increase in DON content of wheat at 20 °C, 25 °C, and 30 °C was significantly accelerated. The DON content in wheat stored at 15 °C was measured at 354.12 μg/kg after storage, with reductions of 22.37%, 39.49%, and 45.71% for wheat stored at 20 °C, 25 °C, and 30 °C, correspondingly.

ZEN is a fungal-derived nonsteroidal estrogenic mycotoxin [35]. Figure 3D shows an initial ZEN content of 6.15 μg/kg in wheat grains. During the 0–60 d storage period, the ZEN content in wheat remained relatively stable. Thereafter, it increased rapidly by day 90. After 180 d of storage at 15 °C, the ZEN concentration in wheat grains reached 10.59 μg/kg, which was 0.66 μg/kg, 1.77 μg/kg, and 4.62 μg/kg lower than that in grains stored at 20 °C, 25 °C, and 30 °C, respectively.

**Figure 4:**
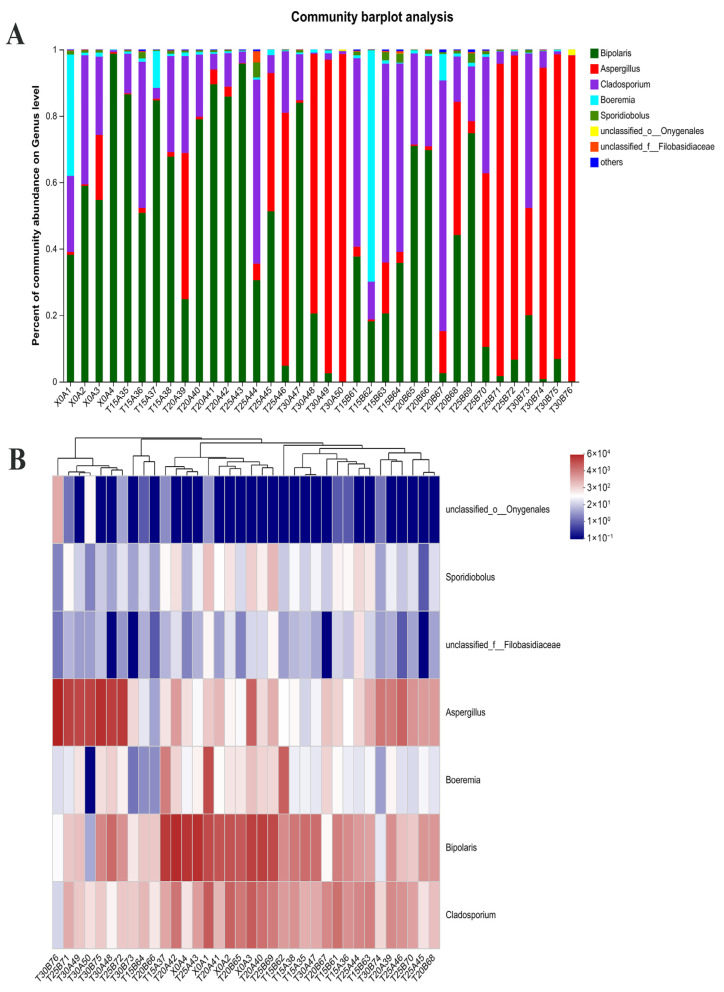
Species richness of wheat fungi at the genus level in different treatment groups (**A**), and heatmap of species cluster analysis in the top seven of relative abundance (**B**). Storage temperature is coded as T, where T15, T20, T25, and T30 denote 15 °C, 20 °C, 25 °C, and 30 °C, respectively. Wheat storage time is coded as A and B. The wheat samples are designated as follows: A1 to A4 for the four samples at 0 d, A35 to A50 represent the 16 wheat samples with four different moisture contents (12.5%, 13%, 13.5%, and 14%) each stored under four temperature conditions (15 °C, 20 °C, 25 °C, and 30 °C) for 90 d, and B61 to B76 represent the 16 wheat samples with four different moisture contents (12.5%, 13%, 13.5%, and 14%) each stored under four temperature conditions (15 °C, 20 °C, 25 °C, and 30 °C) for 180 d.

The species richness of wheat fungi at the genus level under different temperature treatment groups of 0 d, 90 d, and 180 d was analyzed. As shown in Figure 4A, *Bipolaris* was the sole dominant genus in wheat (X0A4) during initial storage (0 d), with a relative abundance reaching 98.68%. After 90 d of storage, wheat under 15 °C (T15A38) and 20 °C (T20A42) conditions was dominated by *Bipolaris*, while wheat under 25 °C (T25A46) and 30 °C (T30A50) conditions was dominated by *Aspergillus*. After 180-day storage, wheat (T20A42) at 15 °C was dominated by *Cladosporium*, while wheat (T20B68) at 20 °C showed *Bipolaris* dominance. At 25 °C (T25B72) and 30 °C (T30B76), *Aspergillus* prevailed in wheat, reaching peak abundance (98.08%) at 30 °C.

A heatmap of species cluster analysis (Figure 4B) was employed to analyze the predominant fungal genera and microbial community dynamics in wheat samples, clearly displaying their abundance variations. In wheat samples with 14% moisture content, samples exhibiting >80% relative abundance of *Aspergillus* were as follows: wheat stored at 30 °C for 90 d (T30A50), wheat stored at 25 °C for 180 d (T25B72), and wheat stored at 30 °C (T30B76).

### 3.3. Principal Component Analysis (PCA) and α-Diversity Analysis

PCA based on distances between samples from different treatment groups revealed differences and changes in species composition among environments. The confidence ellipses represent ‘true’ samples at 95% confidence level. ‘PC1’ and ‘PC2’ denote the first and second principal components, respectively. *α*-diversity analysis measures within-sample microbiota diversity, reflecting community richness and diversity. Operational taxonomic units (OTUs) were clustered at 97% similarity, followed by rarefaction curve construction.

## 4. Discussion

### 4.1. Quality Changes in Wheat During Storage

Lipids and their oxidation products function as signaling molecules during wheat quality deterioration under unfavorable storage conditions [36]. Udayakumar et al. demonstrated a positive correlation between wheat fatty acid values and both moisture content and storage duration [37]. In this study, wheat stored at 15 °C, 20 °C, and 25 °C exhibited significantly smaller increases in fatty acid values compared to 30 °C, suggesting that lower storage temperatures effectively delay fatty acid accumulation. Following 12-month storage at 25 °C, wheat unit weight declined from 812.3 kg/m^3^ to 773.3 kg/m^3^ [38]. These findings align with this study’s results, likely due to enhanced wheat respiration at elevated storage temperatures promoting organic matter degradation and consequent dry matter loss [39]. Strelec et al. demonstrated that low-temperature storage effectively suppressed wheat grain respiratory metabolism and oxidative deterioration, maintaining germination rates above 97% after 360 d [40]. This indicates that low temperatures effectively maintain wheat seed germination capacity. Wheat storage induces physiological enzyme activity alterations. Declining enzymatic activities disrupt normal metabolic functions, resulting in metabolic imbalance. ROS over accumulation-induced oxidative damage primarily drives wheat quality deterioration during storage. As the predominant ROS form, the strong oxidant H_2_O_2_ causes cellular damage when excessively accumulated in wheat tissues. CAT is a key enzyme for removing hydrogen peroxide [41]. Jin et al. demonstrated that low-temperature plasma pretreatment markedly increased CAT activity in corn kernels [42]. Wheat with 14–16% moisture content exhibited progressively declining CAT activity during room temperature storage compared to freshly harvested samples [43]. In this study, the CAT activity level of wheat stored at 15 °C for 180 d was the highest, indicating that a low-temperature storage environment is conducive to maintaining CAT activity and eliminating ROS accumulated during wheat storage to delay quality deterioration. Wheat grain coloration primarily depends on bran pigments, particularly anthocyanins and flavonoids [44]. At 30 °C, wheat b* values showed significant increases after 90-day storage, aligning with Millati et al. who reported high-temperature-induced yellowing in brown rice [45]. Kibar et al. reported that during 360-day storage, quinoa at 25 °C exhibited significant L* value reduction alongside increased a* and b* values, whereas minimal color changes occurred at 4 °C [46]. Meanwhile, this study found that wheat stored at 15 °C and 20 °C showed obviously smaller color changes than at 25 °C and 30 °C, confirming that low temperatures help maintain wheat color stability. Therefore, low-temperature storage preserves the color and quality of wheat by reducing ROS and FAV, thereby enhancing seed viability and unit weight.

The gelatinization properties of wheat starch influenced the quality of end products such as bread, noodles, and steamed buns [47]. For instance, peak viscosity played a critical role in determining the texture and flavor of noodles [48]. Nickhil et al. revealed altered pasting properties in ozone-treated chickpea particles. In this study, wheat stored at 15 °C, 20 °C, and 25 °C showed lower pasting properties than those stored at 30 °C [49]. Similarly, Paraginski et al. demonstrated that storage temperature significantly influences the pasting properties of wheat starch [50]. Thus, the pasting properties of wheat starch are influenced not only by starch structure and processing methods but also by storage temperature. Meanwhile, elevated temperatures markedly reduce gluten’s water absorption capacity by accelerating gluten aggregation, promoting protein cross-linking, and enhancing starch–water competition [51]. This indicates that the water absorption rate of wheat gluten is also affected by temperature. Previous studies demonstrate that α-amylase hydrolyzes wheat starch, accelerates germination, and adversely affects processing quality of wheat flour [52]. Struyf et al. provided complementary evidence showing that elevated α-amylase activity in wheat causes the dough to develop a sticky texture and a darkened appearance [53]. In the initial storage stage, α-amylase activity increases with temperature. Thus, the excessively high α-amylase activity of wheat during storage is one of the key factors leading to the deterioration of its quality. Therefore, a low temperature maintains the processing quality of wheat by delaying the deterioration of starch gelatinization properties, mitigating the reduction in gluten water absorption capacity, and sustaining the suppression of α-amylase activity.

### 4.2. Fungal Spoilage Dynamics in Wheat During Storage

During storage, grain mold contamination is typically assessed through total mold colony counts. For wheat stored at moisture contents of 16%, 18%, 20%, and 22%, the total fungal count was positively correlated with storage temperature [54]. This is consistent with this study’s results, but at 15 °C, the growth of mold numbers was significantly inhibited (Figure 3A). Notably, the total mold colony counts in wheat stored at 15 °C, 20 °C, and 25 °C remained below the threshold of 4 lg CFU/g throughout the 180 d storage period. Mycotoxins represent secondary metabolites generated by diverse fungal species [55]. Hassane et al. reported that AFB_1_ accumulation in 14% moisture wheat flour reached maximum levels at 25 °C when stored for 15 d, compared to 20 °C and 30 °C conditions [56]. This finding is consistent with the present study’s 0–90 d storage result. Notably, extended storage to 120–180 d resulted in maximal AFB_1_ content in wheat at 30 °C. Yi et al. reported that the DON content in uninfected wheat fluctuated within 50 μg/kg during storage, remaining near the initial level of 46 μg/kg [57]. In contrast, corn weevil-infected wheat exhibited significant DON variation with prolonged storage. Erazo et al. confirmed that wheat stored at 15 °C contained the lowest DON levels [58]. Zhang et al. reported that 25 °C may be the optimal temperature for the production of DON [59]. The present study showed significantly lower DON levels in wheat stored at 15 °C for 180 d compared to the other three temperatures (Figure 3B), demonstrating that wheat DON content depends not only on pest infestation but also storage temperature. An increase in ZEN content was observed in wheat after storage at four different temperatures for 180 days. Similarly, Ingram et al. reported an optimal temperature range of 25–30 °C for ZEN production in wheat [60]. In addition, warm temperatures (20–28 °C) and cooler conditions (15–17 °C) both promoted ZEN production by *F. graminearum* and *Fusarium culmorum*. Overall, low temperature was effective in suppressing ZEN production during wheat storage.

Field grains are commonly affected by *Fusarium* and *Cladosporium*, while *Penicillium*, *Aspergillus*, and *Rhizopus* show higher incidence during storage [61]. *Fusarium* predominated in wheat during initial storage, maintaining an incidence rate of 12.5–61.7% [62]. However, prolonged storage reduced *Fusarium* and other genera in wheat, while increasing *Aspergillus* and *Penicillium* levels [63]. Wang et al. observed that low-DON wheat initially contained field fungi (*Alternaria* and *Fusarium*), while storage fungi (*Aspergillus*, *Issatchenkia*, and *Holtermanniella*) increased progressively during storage [64]. After 8 months, *Aspergillus* became absolutely dominant, completely replacing field fungi. This study found that at 15 °C and 20 °C storage temperatures, the dominant fungal genus in wheat shifted from the field-adapted *Bipolaris* to the transitional *Cladosporium*. In contrast, storage at 25 °C and 30 °C led to rapid dominance by the storage-adapted *Aspergillus*. Earlier work by Neme et al. indicated that the severity of fungal deterioration was caused by population diversity and density, which in turn directly relate to temperature [65]. In wheat stored at 25 °C for 180 d, the dominant fungal genus shifted to *Aspergillus*; however, the total fungal count remained below the established threshold (Figure 3A), and no visible mold growth was observed. These results demonstrate that temperature significantly influences fungal genus composition during wheat storage, with lower temperatures inhibiting the growth of storage-type fungi. The relatively abundant fungal genera were analyzed using cluster heatmap analysis, revealing significant differences in dominant genera across samples.

### 4.3. PCA and α-Diversity Analysis on the Fungal Community Composition in Wheat During Storage

**Figure 5:**
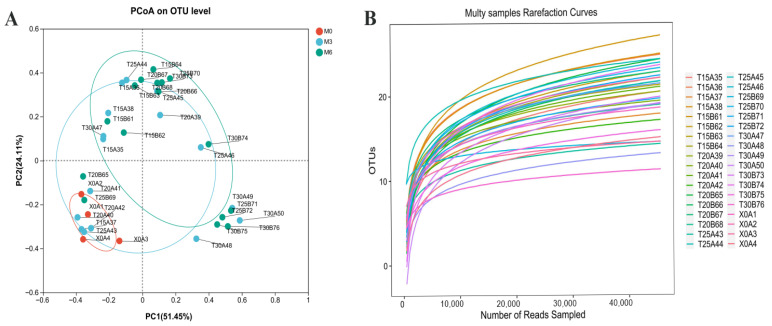
(**A**) Change in total fungi content at different storage temperatures (M0, M3, and M6, respectively represent wheat samples at 0 d, 90 d, and 180 d). (**B**) Rarefaction curves of the stored wheat at different storage temperatures.

Figure 5A presents the PCA results, demonstrating obvious segregation of fungal communities among samples under varying temperature conditions and storage durations. PC1 and PC2 collectively accounted for 75.56%, ensuring sufficient capture of principal component information [66]. Meanwhile, the wheat sample at initial storage (M0) showed the closest proximity to 3-month stored samples (M3) but the farthest distance from 6-month stored samples (M6), indicating the most significant difference in fungal community composition between M0 and M6. Wang et al. observed gradually increasing fungal community diversity with prolonged storage at both 25 °C and 30 °C. Following dataset normalization, alpha diversity was assessed [67]. The rarefaction curve of the wheat microbiota nearly approached asymptotes, as shown in Figure 5B, indicating that the sequencing depth was sufficient to represent the majority of the microbial community variety.

## 5. Conclusions

Low-temperature storage (15–20 °C) effectively maintained wheat quality by significantly inhibiting FAV increase, delaying test weight reduction, preserving germination rate, color and catalase activity, stabilizing flour pasting properties, gluten water absorption, and α-amylase activity, while suppressing mold growth and mycotoxin accumulation (AFB_1_, DON, ZEN). Meanwhile, low temperatures inhibited the succession of wheat fungi from field types (e.g., *Cladosporium*) to storage types (e.g., *Aspergillus*), accompanied by distinct differences in the fungal richness diversity index at different storage temperatures. This work demonstrates the storage quality, processing characteristics, and microbial dynamics in high-moisture wheat (14%) under different storage temperatures, establishing theoretical foundations for safe storage of high-moisture wheat.

## Data Availability

The data presented in this study are available on request from the corresponding author.
